# Olfactory EEG based Alzheimer disease classification through transformer based feature fusion with tunable *Q*-factor wavelet coefficient mapping

**DOI:** 10.3389/fnins.2025.1638922

**Published:** 2025-08-28

**Authors:** Berke Cansiz, Hamza Osman Ilhan, Nizamettin Aydin, Gorkem Serbes

**Affiliations:** ^1^Department of Electronics and Communication Engineering, Yildiz Technical University, Istanbul, Türkiye; ^2^Department of Computer Engineering, Yildiz Technical University, Istanbul, Türkiye; ^3^Department of Computer Engineering, Istanbul Technical University, Istanbul, Türkiye; ^4^Department of Biomedical Engineering, Yildiz Technical University, Istanbul, Türkiye

**Keywords:** Alzheimer's disease, olfactory stimulation, common spatial pattern, covariance matrix-tangent, tunable Q-factor wavelet transform, electroencephalography, transformer-based fusion, mild cognitive impairment

## Abstract

**Introduction:**

Alzheimer's disease has been considered one of the most dangerous neurodegenerative health problems. This disease, which is characterized by memory loss, leads to conditions that adversely affect daily life. Early diagnosis is crucial for effective treatment and is achieved through various imaging technologies. However, these methods are quite costly and their results depend on the expertise of the specialist physician. Therefore, deep learning techniques have recently been utilized as decision support tools for Alzheimer's disease.

**Methods:**

In this research, the detection of Alzheimer's disease was investigated using a deep learning model applied to electroencephalography signals, taking advantage of olfactory memory. The dataset comprises three categories: healthy individuals, those with amnestic mild cognitive impairment, and Alzheimer's disease patients. The proposed model integrates three distinct feature types through a transformer-based fusion approach for classification. These feature vectors are derived from the Common Spatial Pattern, Covariance matrix-Tangent Space and a Tunable Q-Factor wavelet coefficient mapping.

**Results:**

The results demonstrated that subject-based classification of rose aroma attained a 93.14% accuracy using EEG-recorded olfactory memory responses.

**Conclusion:**

This output has demonstrated superiority over EEG-based results reported in the literature.

## 1 Introduction

Alzheimer's Disease (AD), the prevalent form of dementia, is a neurodegenerative disorder characterized by progressive cognitive decline and memory deficits (Gold and Budson, 2008), predominantly affecting older adults (Castro et al., 2010). Neuronal loss, accumulation of amyloid beta (Aβ) proteins, and alteration of tubulin associated unit (TAU) protein have been identified as the cause of the disease (Paula et al., 2009). Specifically, it has been revealed that the detrimental effects of Aβ oligomers on synaptic connections affect the memory system (Koffie et al., 2011). Research findings point out that AD considerably increases mortality risk in addition to causing cognitive deterioration (Mölsä et al., 1986; Burns et al., 1990). AD represents a major healthcare issue due to its adverse impact on life quality and life expectancy. Consequently, early stage diagnosis of Mild Cognitive Impairment (MCI) in Alzheimer's Disease (AD) is crucial to mitigate the adverse effects of the disease. The Mini Mental State Examination (MMSE), one of the common diagnostic instruments for AD, allows for evaluation based solely on test scores, minimizing the necessity for physician expertise; however, it has been noted that differentiating between symptoms of aging and those of MCI can be challenging (Chapman et al., 2016). Another common application is to detect neurologic structural changes through a range of imaging techniques (Mosconi et al., 2007). However, the challenges posed by radiation exposure, procurement challenges (accessibility issues), invasiveness, and the costs associated with devices such as PET present constraints (Yu et al., 2021). This situation encourages the exploration of innovative and accessible approaches for diagnosing AD.

Multiple studies in the literature have established a link between odors and memory (Herz, 2016). An aroma with remnants of the past can permeate the mind and evoke bygone memories even after years. Certain odors could trigger past memories, which could be useful as a diagnostic tool for Alzheimer's disease. Memory impairment resulting from AD leads to a lack of response to familiar odors, as these aromas fail to trigger any cognitive associations. Current research shows that this sensory contrast can be a powerful tool in the diagnosis of AD and MCI (Invitto et al., 2018). Recent studies have identified that neurofibrillary tangles associated with AD result in neuronal loss in the olfactory bulb, suggesting early detection of AD-related olfactory dysfunction is possible (Morgan and Murphy, 2012).

Electroencephalogram (EEG) can be used to measure brain activity stimulated by odor (Kroupi et al., 2014). EEG, which has high temporal resolution, allows the brain's response to stimulation to be measured instantly. The EEG exhibits a complex multi-channel structure characterized by the combined of both high and low oscillations. This complex structure shows the electrical activity that occurs as a result of communication between neurons in the brain as a voltage change. Different types of oscillation characteristics provide information about the brain's working function. Sedghizadeh et al. (2020) documented that AD induces a reduced synchronization of high-frequency oscillations in the frontal cortex, suggesting a potential diagnostic marker. For this reason, the mental activity to be stimulated by odor can be monitored with an EEG device. The EEG-based approach is notable for being invasive and avoiding radiation (Lazarou et al., 2018). Machine learning techniques can be used to identify variations in changes between individuals.

This study proposes a model using olfactory EEG signals to detect Alzheimer's disease (AD) and early mild cognitive impairment (MCI). In the model configuration, spatial, temporal, and spectral information was extracted from the EEG data. To acquire this information, approaches for signal processing and analysis have been utilized, including the common spatial pattern (CSP), the tangent space matrix covariance and the tunable *Q*-factor wavelet transform (TQWT). By integrating multiple feature types (CSP for spatial patterns, covariance for global brain dynamics, and TQWT for time-frequency details), our method leverages complementary information; this multi-faceted approach is intended to capture AD-related EEG alterations more comprehensively than simpler single-domain methods. The study expected that superior classification performance might be achieved by combining various feature information, leading to the establishment of a transformer-based fusion model. To the best of our knowledge, this is the first work that combines olfactory–evoked EEG signals with transformer–based multimodal feature fusion for three–class staging of Alzheimer's disease.

The contributions of the study are listed as follows:

- A robust model configuration has been implemented where spatial information of the EEG signal and time-frequency analysis are fused at the feature level.
- A novel model has been established that allows time-frequency and spatial analysis. In this approach, tokenization is executed for each sub-band of the signal, hence providing importance to each sub-band in olfactory EEG analysis.
- Wavelet Coefficient Mapping (WCM) will be performed for the first time with the tunable *Q*-factor wavelet transform (TQWT) to evaluate the performance of Alzheimer's classification through the olfactory EEG signal.

The rest of the paper is organized as follows; Section 2 presents the information of related studies. The dataset used in the study and detailed information about the methods implemented are described in Section 3. The experimental results are given in Section 4. The discussion of the experimental results is provided in Section 5. Finally, the study is concluded in Section 6.

## 2 Related studies

In the diagnosis of AD, a range of sensory-based psychophysical tests are utilized, with various studies in the literature addressing vision (Salobrar-Garcia et al., 2015), hearing (Tuwaig et al., 2017), and olfaction. A study on the olfactory system (Arnold et al., 2010) revealed that the AD group had Aβ accumulation in the olfactory receptor. This indicates that olfactory perception is important in diagnosing AD, with diminished odor serving as a potential signal of the disease. Olfactory assessments can be employed in this setting to identify sensory deficits. In one of the study (Wang Q. S. et al., 2002), it is indicated that olfactory function typically declines in early-stage MCI patients, and its assessment may facilitate the early diagnosis of AD. They employed the cross-cultural variant of the smell identification test devised by Doty et al. (1984). The research conducted by Growdon et al. (2015) revealed that neurodegenerative alterations associated with AD and the distinction between AD patients and healthy individuals could be discerned by olfactory recognition assessments in a cohort of 215 participants. However, as these evaluations are based on participant input, the outcomes may be susceptible to deceptive influences. Furthermore, the habituation stage, characterized by a decrease in perception after long-term exposure to an odor, can produce undesirable results during the assessment (Wang L. et al., 2002). Consequently, the application of EEG-based Olfactory Event Related Potential (OERP) techniques, which exhibit gradual adaptation to olfactory stimuli and remain operational despite perceptual loss, can address this problem (Wang et al., 2004). OERP, recognized as the favored methodology in various investigations, has facilitated the analysis of EEG signals evoked by olfactory stimuli (Arpaia et al., 2022). Hyposmia, an early impairment in odor perception, can be identified by OERP (Brämerson et al., 2008). In a study employing this approach, a reduction in amplitude associated to AD was noted in the N1 component, a distinctive wave of the OERP and the initial negative polarized response to stimulation (Invitto et al., 2018).

Although OERP provides a generally powerful analysis opportunity for EEG signals, obtaining it by averaging the number of experiments is impractical for methods that require a large number of samples, such as machine learning. For this reason, there are studies in the literature that examine the signals of EEG experiments. In one of these studies (Tawhid et al., 2023), the objective was to find the most successful band range to classify healthy subjects with MCI. Initially, all bands of the EEG signals were filtered and separated, followed by construction to spectrogram images for each respective band. Then, the classification process was performed using 2D CNN. Upon examination of the classification results, it was observed that the images belonging to the beta band gave the strongest classification performance. Theta and alpha were reported to emerge as the most effective bands following the beta band. It was also observed that the band that exhibited the least success was delta. In another study (Nayana et al., 2025), spectrogram images constructed for each channel were stacked sequentially and classified with a 2D CNN model. The evaluation of classification involved three datasets. For the categories AD, frontotemporal dementia (FTD) and CN, 2D CNN demonstrated an accuracy, precision, recall, and F1-score ranging from 91.1% to 91.92%. Meanwhile, in the classification of CN, MCI, and AD, the scores were between 86.25% and 87.61%, maintaining consistency across the same metrics. In another study (Olcay et al., 2025), researchers performed sub-band analysis and extracted various features from statistical energy variations prior to and after the stimulus. They tested these features with various machine learning methods. In their results, high accuracy performance was obtained as 87.5%. In addition, it was observed that the performance enhance notably in the scenario where MMSE results were integrated using a multi-modal approach.

## 3 Materials and methods

This section provides a detailed explanation of the dataset utilized in the study, followed by subsections that explain the proposed approach, including signal processing methodologies, feature fusion, and classification.

### 3.1 Dataset description

Olfactory EEG dataset created by Sedghizadeh et al. (2023) was utilized in this study. Data were collected from a group including individuals with Alzheimer's Disease, Mild Cognitive Impairment, and cognitively healthy controls. The group consists of 35 seniors, including 13 diagnosed with AD, 7 with MCI, and 15 healthy individuals with total of 3,877 samples. Two separate aroma types were involved in the study, leading to the creation of two independent classification sets. Of the two aromas, lemon was emitted more often (75% probability) compared to rose (25% probability). Each participant received randomized trials of rose and lemon, with fixed probabilities (1:3) to prevent long runs of the same odor. Short breaks were given between trials to allow the odor to dissipate and to avoid fatigue or habituation. During 120 trials, a 2-second odor release was followed by an 8-second rest period. EEG data was collected from the Fp1, Fz, Cz, and Pz regions in 4 channels at a sampling frequency of 200 Hz. During data collection, researchers removed eye blink using independent component analysis, and signal filtering was performed between 0.5 and 40 Hz (Sedghizadeh et al., 2023).

### 3.2 Signal processing

EEG signals were obtained in a multi-channel structure obtained from various electrodes with non-stationary high and low oscillations. This complex structure can be examined with various spatial, temporal and spectral analysis methods. In this section, the description of utilized signal processing methods is given.

#### 3.2.1 Spatial analysis

Analyzing spatial correlations across various channels is beneficial due to the shared information anticipated to be derived from them. One approach, known as common spatial pattern (CSP), identifies spatial filters that enhance inter-class sample variance while reducing intra-class sample variance (Wang et al., 2011). The projection matrices obtained with the filters of CSP, which is a supervised method, distinguish the variation difference of two classes. During the implementation, the number of CSP components was set to 4. Covariance matrices were estimated by concatenating all the trials, which provides a more stable estimate across subjects. It should be noted that these filters were calculated on the training data only to avoid data leakage. In addition, regularization was not employed during the implementation. The transformed components are extracted as the average power of each spatial filter. Another spatial analysis method, Covariance Matrix Tangent Space (CMTS), obtains the covariance matrix for the examination of the relationship between channels. This covariance matrix is in the Symmetric Positive Definite (SPD) matrix structure and is defined in a curved space defined as a Riemann manifold. Tangent curves are extracted with the logarithmic mapping method performed on these SPD matrices. In this way, the matrix structure in the Reimann space is transformed into a vector structure in the Euclidian space. Thus, a linear feature vector is obtained.

#### 3.2.2 Time-frequency analysis

The analysis of EEG presents significant challenges due to its complex and overlapping patterns of high and low oscillations. Low oscillation necessitates high spectral resolution over extended periods, while high oscillation, characterized by transient (short duration), demands high temporal resolution for short intervals. This level of adaptability is unfeasible in techniques like the Short Time Fourier Transform, which utilizes a fixed bin transformation (Cansiz et al., 2025). Consequently, the Wavelet Transform (WT), capable of flexibly extracting frequency and temporal information through a mother wavelet, along with its scaling and shifting characteristics, has emerged as a significant option. There are two different types of WT methods, namely discrete and continuous, and the discrete one is more convenient in terms of calculation (Shensa, 2002). Discrete WT (DWT) is characteristically a dyadic transform and the output coefficient number and the length of the input signal are equal. DWT applied by selecting a fixed mother-wavelet type does not offer sufficient flexibility in controlling the quality (*Q*) parameter representing the center frequency and bandwidth ratio of relevant sub-band. This approach mitigates the efficacy of robust frequency analysis techniques. Choosing a high *Q* value for high-oscillation components and a low *Q* value for low-oscillation signals is necessary for robust signal analysis (Pachori, 2023). With the Tunable *Q*-factor Wavelet transform (TQWT) introduced in Selesnick (2011), it has become possible to adjust this *Q* value flexibly for each sub-band (Sakar et al., 2019; Cansiz et al., 2024). TQWT, which is an overcomplete transform, is a redundant transform and increases the number of coefficients compared to the dyadic decomposition. This makes it possible to obtain a stronger representation of the time-frequency plane.

The TQWT parameters (*Q*-factor and number of levels *J*) were selected based on a combination of prior literature and empirical validation on our training data. We initially consulted previous studies and observed that in a study (Bhattacharyya et al., 2017) on wavelet analysis of EEG that utilized *Q* in the range of 1 to 4 to capture the oscillatory behavior of EEG. Therefore, during the experiments, several values of *Q* (from 1 to 4) and different decomposition depths (*J* from 3 to 9) were employed.

**Figure 1:**
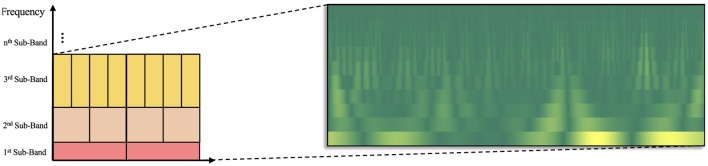
Construction of TQWCM image (the resolution of the time-frequency tiling is controlled by the choice of Q-Factor to obtain the optimum representation of AD, MCI and Healthy subject patterns).

The TQWT function produces wavelet coefficients for each sub-band. Stacking each coefficient enables the creation of time-frequency representation of the signal. However, the coefficients belonging to a bin having high time resolution represents a shorter width in time axis (results in a bigger height in frequency axis due to fixed area of each bin on time-frequency tiling), while the coefficients belonging to a bin having high frequency resolution represents a larger width in time axis (results in a smaller height in frequency axis). This actually disrupts the equality of the time-frequency tiling distribution on the time and frequency axes due to the variability of scale factor. Consequently, linear interpolation was employed to the distribution of bins, numerically equalizing all bands. The scalogram-like visual produced in this manner was achieved by a discrete transformation. Figure 1 illustrates the Tunable *Q*-Factor Wavelet Coefficient Mapping (TQWCM) visualization.

### 3.3 Feature extraction

In the remainder of Section 3, we detail the two complementary stages of our feature-extraction pipeline. Section 3.3.1 describes the convolutional network that converts each Tunable-Q Wavelet-Coefficient Map (TQWCM) into a compact set of image-based features, capturing both long- and short-term time-frequency patterns. Section 3.3.2 then explains how these TQWCM features are fused with spatial (CSP) and connectivity (CMTS) descriptors by means of a lightweight transformer encoder, enabling the model to learn cross-modal relationships before classification.

#### 3.3.1 Feature extraction from TQWCM

**Figure 2:**
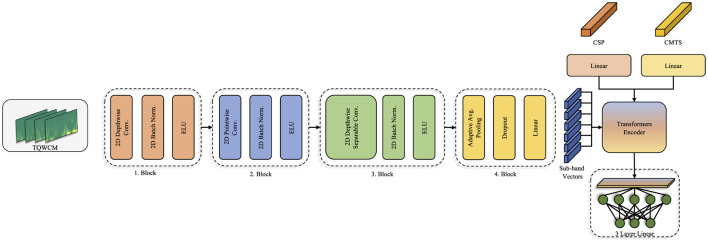
Classification model architecture.

A specialized convolutional network was developed for extracting features from the generated TQWCM images, incorporating various depthwise and pointwise layers, similar to those used in the EEGNet (Lawhern et al., 2018) model. Differently, since it was fed as a TF image during the application, EEG channel information was used as visual channel information. Thus, while the depthwise layer performs intra-channel time frequency analysis, the relationship between channels is evaluated with the pointwise layer (Chollet, 2017). During implementation, the number of channels in the depthwise layer was the same as the number of EEG channels, and the kernel size was set as (1,64) with (1,32) padding. In the pointwise layer, number of channels was increased to 64 with (1,1) kernel size. These two layers are repeated with a depthwise separable block with a smaller filter size (1,16) in the next step. With the block structure created with these two different width filters, both long-term and short-term temporal relationships are revealed in detail. Regarding to previous blocks, number of channels was fixed in the depthwise layer and increased to 128 in the pointwise layer. The constructed model architecture is depicted in Figure 2.

#### 3.3.2 Transformer based fusion

The transformer model (Vaswani et al., 2017), known for its effectiveness in large language models, provides a relationship between words and their contribution to the overall context through multi-headed attention processes derived from inputs as tokens. These tokens evaluate the significance of information within the attention process. This token format is believed to facilitate the investigation of the links between characteristics and is preferred for the fusion of characteristic levels in many studies (Huang et al., 2020; Roy et al., 2023; Wang et al., 2022). Within the scope of this study, the aim is to integrate spatial and time-frequency analysis characteristics to leverage their fusion and acquire robust insights on the signal. Consequently, tokens are generated for CSP, CMTS, and each sub-band detail within the constructed model. The Transformer structure improves the transfer of information between features.

In our transformer-based model, we treat different feature sets as separate input tokens (e.g., a token for CSP features, tokens for each covariance feature, tokens for each TQWT sub-band feature). This design allows the self-attention mechanism to weigh information from different sources. The intuition is that each feature type provides complementary information—for instance, CSP features capture discriminative spatial patterns of oscillatory power, covariance (CMTS) features capture overall connectivity patterns, and TQWT features capture time-frequency dynamics. By feeding them as distinct tokens, the transformer can ‘decide' how much attention to pay to each source for a given classification decision. This is in contrast to simply concatenating all features into one long vector. The tokenized approach with self-attention can potentially model complex interactions between feature types and focus on the most informative ones for distinguishing between classes.

### 3.4 Experimental procedure

In the experiment, the first phase entailed dividing the dataset into training and test sets through leave-one-subject-out (LOSO) methodology. In each fold, patient-specific samples formed the test set, with the rest allocated for training. This approach guarantees that models are trained on preceding patients before being applied to a new patient and eliminates data leakage by keeping individual patient's data out of the training set. LOSO was chosen due to the small dataset, ensuring that no subject's data is used in both training and testing, thus providing an unbiased estimate of model performance on new subjects.

**Figure 3:**
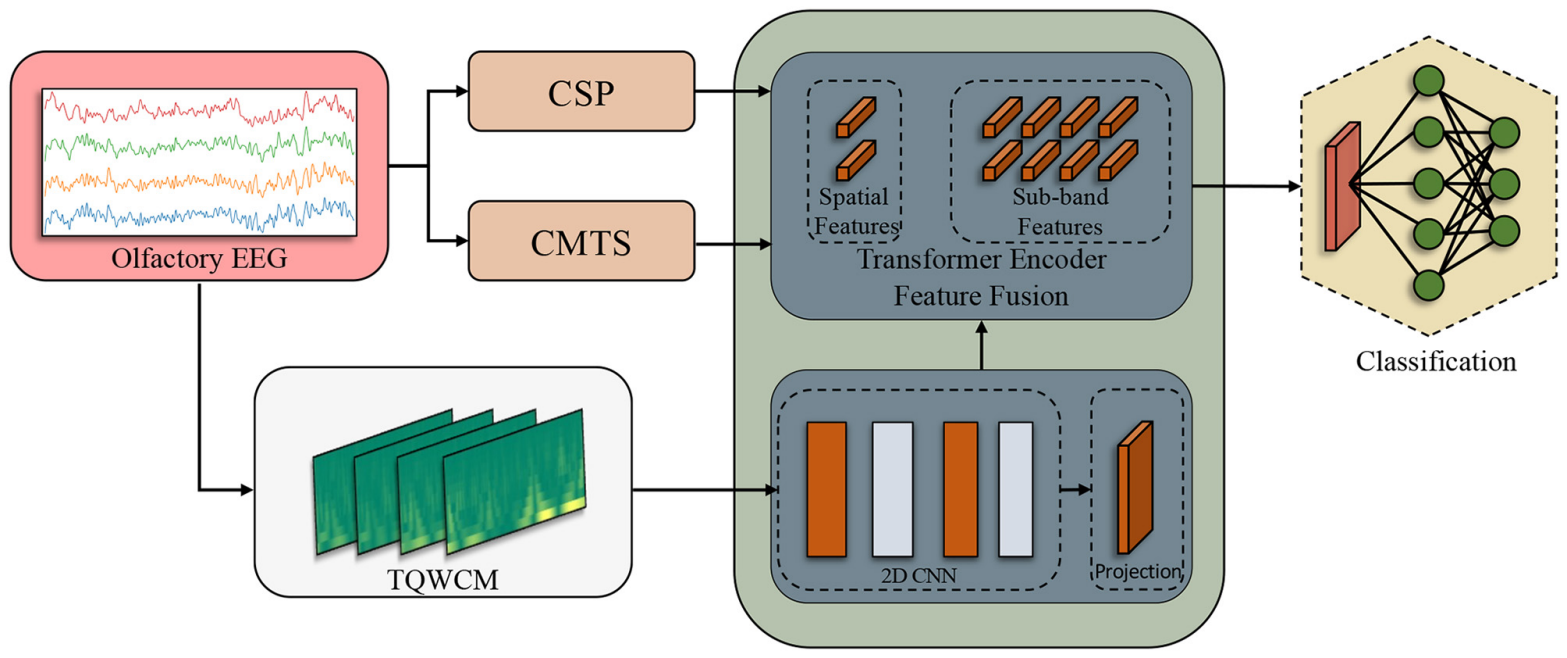
Workflow of the olfactory EEG-based Alzheimer disease classification model.

Subsequently, the signal underwent processing through the spatial analysis techniques CSP and CMTS. With CSP, feature vectors were generated corresponding to the number of channels, which was four in this study. The Covariance Matrix (CM) was obtained using the orthogonalized analytical shrinkage method. Then, Tangent Space (TS) was applied on this matrix. In order to prevent any data leakage during the CSP and TS application, it was fit with training and then transformed separately. Alongside these processes, the time-scale coefficients were derived using the TQWT applied on raw signals. The coefficients produced distinct TQWCM images for each channel, organized in a three-dimensional format that includes channel, frequency, and time. The model was sequentially fed the acquired inputs. As the initial layers handled the TQWCM image, the middle layers integrated all three feature types. In the process of tokenization, CSP and CMTS represented each token individually, whereas the sub-band information of TQWCM represented the tokens in an independent way. The total count of tokens generated was established as 2 + (the total number of sub-bands). At the end of this process, the transformer encoder output fed the 3-layer linear structure and produced the output. The comprehensive workflow is illustrated in Figure 3.

During the implementation, the model was trained 50 epoch along with a batch size of 64, and the Adam optimizer was employed with a learning rate of 0.001. In addition, the model was incorporated dropout regularization in the convolutional blocks (with rate 0.3) and linear layers (with rate 0.5).

## 4 Results

Section 4 presents the empirical evaluation of the proposed framework. Section 4.1 introduces the employed classification metrics with leave-one-subject-out cross-validation. Section 4.2 analyses general classification performance for different TQWT parameters and odor types.

### 4.1 Evaluation metrics

During the evaluation of the model results, LOSO method was applied and all signal data associated with a subject were designated as test data, while the remaining individuals were organized as training data. Consequently, a comprehensive set of 35 evaluations was performed for each patient in an individual manner. In addition, this fold structure was established through 5 repetitions and average scores were presented. This contributed to the reliability of the model evaluation metrics and more generalized results were presented. The results obtained according to the highest test scores during training were analyzed using various metrics. Among these metrics, accuracy, which shows the general correct prediction success, precision, which provides analysis opportunity for unbalanced datasets, recall and F1-score, which represents the harmonic average of both, were utilized. Especially in problems with an unbalanced class distribution such as the employed dataset, considering other metrics in addition to accuracy is crucial for model evaluation (Sokolova and Lapalme, 2009). Although the dataset is highly unbalanced, we chose not to apply synthetic oversampling (e.g., SMOTE) or class weighting in the loss function for this study. Instead, we relied on performance metrics beyond accuracy (precision, recall, F1) to evaluate any class bias. Given the small sample, oversampling techniques might have risked producing overfitted models (by effectively replicating rare class samples), and the LOSO scheme ensured every class member is eventually tested. Therefore, we opted to train the model on the data distribution as-is, but we carefully monitored class-wise performance.


(1) $$\begin{array}{l} \text{Accuracy}=\frac{TP+TN}{TP+TN+FP+FN} \end{array}$$

(2) $$\begin{array}{l} Precision=\frac{TP}{TP+FP} \end{array}$$

(3) $$\begin{array}{l} Recall=\frac{TP}{TP+FN} \end{array}$$

(4) $$\begin{array}{l} \text{F1-Score}=2\times \frac{Precision\times Recall}{Precision+Recall} \end{array}$$

During the calculation of the results, the voting operation was performed with the signals belonging to the subject, which were set as the test set. Consequently, the class most predicted for that person was selected as the model prediction. In case of a tie in the voting, the person was determined as AD.

### 4.2 Experimental results

The average performance results, evaluated in the test set after the training procedure, are presented in Table 1. The results are presented with accuracy, precision, recall and F1-score and standard deviation depending on each TQWT parameter under two main odor headings. When examined in general, it is observed that the most successful output in terms of classification performance is obtained with 93.14% accuracy with rose odor. It is accompanied by 90.86% accuracy with lemon odor.

**Table 1:** Average evaluation metrics (%) of the applied method (highest scores are in bold).

**TQWT parameters**	**Lemon**				**Rose**			
	**Accuracy**	**Precision**	**Recall**	**F1-Score**	**Accuracy**	**Precision**	**Recall**	**F1-Score**
*Q*:1, *J*:5	0.8743 (±0.059)	0.8996 (±0.059)	0.8862 (±0.058)	0.8877 (±0.059)	0.8914 (±0.037)	0.9131 (±0.046)	0.9053 (±0.031)	0.9047 (±0.037)
*Q*:1, *J*:9	0.9029 (±0.026)	0.9109 (±0.032)	0.9132 (±0.032)	0.9112 (±0.031)	**0.9314 (±0.026)**	**0.9424 (±0.032)**	**0.9405 (±0.022)**	**0.9394 (±0.028)**
*Q*:2, *J*:5	0.8914 (±0.042)	0.9094 (±0.032)	0.9053 (±0.038)	0.9014 (±0.039)	0.8800 (±0.055)	0.9095 (±0.041)	0.8937 (±0.049)	0.8888 (±0.058)
*Q*:2, *J*:9	0.9029 (±0.043)	0.9114 (±0.045)	**0.9183 (±0.038)**	**0.9132 (±0.043)**	0.8629 (±0.031)	0.8942 (±0.025)	0.8759 (±0.026)	0.8765 (±0.033)
*Q*:3, *J*:5	**0.9086 (±0.024)**	**0.9168 (±0.017)**	0.9168 (±0.028)	0.9128 (±0.024)	0.8857 (±0.029)	0.9199 (±0.028)	0.8981 (±0.025)	0.8978 (±0.03)
*Q*:3, *J*:9	0.9029 (±0.033)	0.9266 (±0.017)	0.9091 (±0.021)	0.9114 (±0.016)	0.9029 (±0.033)	0.9280 (±0.026)	0.9142 (±0.03)	0.9132 (±0.035)
*Q*:4, *J*:5	0.8857 (±0.088)	0.9053 (±0.086)	0.9009 (±0.075)	0.8992 (±0.082)	0.8914 (±0.042)	0.9211 (±0.016)	0.9032 (±0.039)	0.8997 (±0.037)
*Q*:4, *J*:9	0.8914 (±0.051)	0.8987 (±0.044)	0.9053 (±0.044)	0.8969 (±0.045)	0.8400 (±0.026)	0.8864 (±0.031)	0.8571 (±0.023)	0.8482 (±0.031)

Standard deviation of each metric is given in parentheses.

The impact of parameter adjustments on the TQWT results can be inferred by analyzing these results. Consequently, when the outcomes of the odors are analyzed individually; it is observed that 90.86% accuracy and 91.28% F1 score are obtained in lemon odor by using *Q*:3 *J*:5 parameter-set, followed by 90.29% accuracy and 91.32% F1 score obtained with *Q*:2 *J*:9 parameter-set. It is observed that the lowest score in this odor is obtained in *Q*:1 *J*:5 parameter-set as 87.43% accuracy and 88.77% F1 score. In the rose odor, it was observed that 93.14% accuracy and 93.94% F1-score were obtained in the *Q*:1 *J*:9 parameter-set, while the lowest score was 84% accuracy and 84.82% F1-score achieved by usage of the *Q*:4 *J*:9 parameter-set. According to the findings, it is evident that in the lemon odor, the response characteristics of the stimulus become more pronounced at elevated *Q* values, whereas in the rose odor, the reduced *Q* values further increase the differences in class variation.

Table 2, which includes class-based analyses, shows the best classification performance results for both odors. It is observed that the MCI class clearly stands out from the other classes for both lemon and rose odors. The highest classification performance was observed for rose (Q:1, J:9), with precision recall and F1 score results of 97.5%, 100%, and 98.67%, respectively. The worst results were observed for the healthy class: 94.85%, 81.54%, and 87.59%, respectively.

**Table 2:** Class-wise precision, recall, and F1 scores for the best hyperparameter settings on both odor datasets. standard deviation of each metric is given in parentheses.

**Class**	**Lemon (Q:3, J:5)**			**Rose (Q:1, J:9)**		
	**Precision**	**Recall**	**F1-Score**	**Precision**	**Recall**	**F1-Score**
AD	0.8805 (±0.048)	0.9600 (±0.033)	0.9177 (±0.031)	0.9013 (±0.026)	0.96 (±0.033)	0.929 (±0.013)
Healthy	0.9485 (±0.042)	0.8154 (±0.038)	0.8759 (±0.027)	0.951 (±0.040)	0.8615 (±0.058)	0.9026 (±0.035)
MCI	0.9214 (±0.065)	0.9714 (±0.057)	0.9448 (±0.053)	0.975 (±0.050)	1 (±0.000)	0.9867 (±0.027)

## 5 Discussion

AD is a prevalent and deadly type of dementia that significantly diminishes individuals' quality of life. Early diagnosis is crucial for treatment and reduction of the adverse effects of the disease. Our study intended to diagnose AD by analyzing the EEG responses of elderly subjects to olfactory stimuli, as olfactory dysfunction frequently manifests early in AD and MCI. Moreover, specific odors were applied to elderly patients, as they were believed to evoke past memories. The most important motivation of the study was to diagnose AD using only EEG signals instead of tests such as MMSE that could provide misleading information. The analysis revealed the successful attainment of this objective, with the model exhibiting an average classification accuracy of 93.14% (±2.6) through cross-validation and repeated assessments. Moreover, considering that the dataset has an unbalanced distribution, it is important to interpret this success with other metrics. The model demonstrated a distinction between classes with 94.24% (±3.2) recall and 94.05% (±2.2) precision, indicating high consistency in correct class predictions during the evaluation.

Recent work employing only spectral or spatial features with conventional classifiers such as support–vector machines or Linear Discriminant Analysis has achieved accuracies in the 80%–90% range on EEG datasets of comparable size (Pirrone et al., 2022; Kim et al., 2023). While attractive for their algorithmic simplicity, these single–domain approaches may overlook complementary information contained in spatial and time–frequency patterns. Our study addresses this gap by fusing spatial (CSP), network–level (CMTS) and time-frequency (TQWT) representations within a lightweight transformer encoder, allowing cross–modal interactions that have not, to our knowledge, been leveraged in earlier olfactory–EEG research. We believe that this integrated design underlies the superior 93.14% accuracy obtained here despite the small sample size.

The results can be analyzed on the basis of odor types. In the experiment, participants were exposed to two odor varieties, lemon and rose, each with a defined likelihood. The signals formed in the brain in response to these stimuli were measured with an EEG device. Analyzing these signal structures determined that employing the rose aroma yielded the most robust results. This may be due to multiple factors, primarily that the rose odor serves as a stronger marker for AD than the lemon odor. Previous studies have indicated that floral-based odor, particularly roses, tends to cause mainly impairment of recognition with early-stage AD (Umeda-Kameyama et al., 2017). Furthermore, the distinct and strong characteristics of citrus aromas, such as lemon, likely facilitated easy differentiation. Citrus stimuli (lemon) often co-activate trigeminal pathways, potentially introducing variability or habituation, whereas some floral odors (rose) may produce more purely olfactory limbic engagement; this could partly explain the higher accuracy we observed for rose trials, though the literature does not yet provide direct comparative evidence in AD cohorts. Replication with intensity- and trigeminality-matched odors is needed. Another one possible conclusion is that the odor of the rose is emitted less often than that of the lemon. The constant secretion of the lemon odor may have caused a decrease in smell discrimination after a while.

Another factor to consider is cultural familiarity or significance of the odors. All participants in this study were from a similar cultural background (Middle Eastern, specifically Iranian, context). Rose scent (e.g., rosewater) has traditional uses in this culture, but is perhaps encountered less frequently on a daily basis than lemon (which is a common flavor/scent in food and cleaners). The novelty or salience of the rose odor in this cultural context might have led to stronger attention or emotional responses, thereby yielding clearer EEG distinctions. In contrast, the lemon odor, being very familiar, might not engage participants as much or might cause faster habituation.

**Figure 4:**
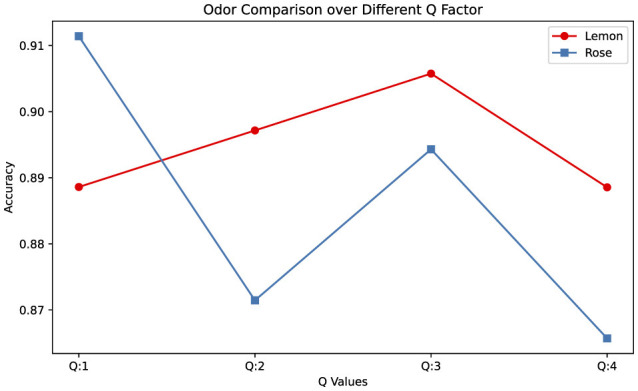
Odor type comparison across different *Q* factor (Average values were calculated across the results of different sub-bands at that *Q* value.

It has also been observed that odors exhibit different characteristics depending on the *Q* parameter of TQWT. Accordingly, EEG measurements in response to lemon odor can be analyzed with high performance with high *Q* parameter, while rose odor can be analyzed with higher performance with low *Q* (as demonstrated in Figure 4). This shows that the response to lemon odor is more noticeable in high oscillations in EEG, while rose odor is more noticeable in low oscillations.

**Figure 5:**
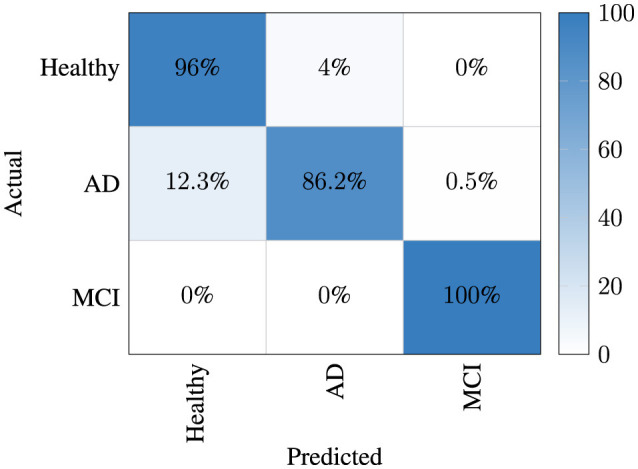
Confusion matrix of highest result of proposed model.

In the classification results, the best average confusion matrix for each trial is given separately for both aromas in Figure 5. It was observed that the MCI class was separated from the other two classes with high performance. Thus, it was determined that the signal structure of the MCI case had a different variation compared to the others and the presented model was able to successfully capture this characteristic. However, when interpreting this figure, one can notice that the model has experienced confusion between the AD and healthy classes.

The comparison of the results with existing studies utilizing EEG in the literature clearly demonstrates its superiority as given in Table 3. Consequently, within the context of numerous comparable investigations, the outstanding performance of the 3-class classification (AD, MCI, and healthy) achieved solely with EEG input, without incorporating additional patient-related information such as MMSE, is particularly significant.

**Table 3:** Literature comparison.

**Study**	**Classification problem**	**Accuracy**
Sedghizadeh et al. (2020)	AD/Healthy	79.20%
Miltiadous et al. (2023)	AD/Healthy	77.01%
Huh et al. (2025)	AD/Healthy	81.70%
Olcay et al. (2025)	AD/Healthy	87.50%
Nayana et al. (2025)	AD/FTD/Healthy	91.13%
	AD/MCI/Healthy	87.61%
Proposed method	AD/MCI/Healthy	**93.14%**

The results from other studies are reported for contextual purposes. Although all works utilize olfactory EEG, differences in class definitions, preprocessing, and protocol details mean these figures should be viewed as indicative rather than as strict, head–to–head benchmarks). Bold value indicates the greatest value among the compared results.

Despite the successful results, the study has several limitations. The first of these is that the dataset used belongs to a single culture and the odors are of low variety. Also, experiments were applied to a limited population and limited number of recording channels. Future studies will include recordings with a full 10–20 system montage to incorporate more spatial information, which may further improve reliability and performance. Additionally, a set with more ethnic diversity can be collected. Future investigations could focus on the analysis of signal characteristics, advancing the study further. This requires that extensive research for various signal processing techniques, including denoising and signal enhancement, however it has been shown that successful classification performance is achieved with the TQWT structure without the need for an additional filtering process. A detailed physiological comparison (e.g., odor-specific band power, ERP component analysis) was not performed here and remains an avenue for future work; such analyses, together with controlled matching of odor intensity and trigeminal contribution, will be necessary to determine whether the observed rose–lemon difference reflects underlying neurophysiological mechanisms or stimulus-specific variability. We acknowledge that our results (rose > lemon in classification performance) might not generalize to other cultures with different odor experiences or preferences. Future studies should either use culturally neutral odors or explicitly measure individual familiarity and preference for the odors, to account for this variable.

## 6 Conclusion

Early diagnosis of Alzheimer's, which is a very dangerous and common type of dementia associated with loss of memory and consciousness, is crucial to reduce the negative effects of the disease. Various mental state tests, neuroimaging, and neurophysiological methods are applied in the early diagnosis of this disease. This study focuses on the detection of Alzheimer's disease using olfactory EEG signals. In olfactory EEG, participants are subjected to various odors at certain intervals. The physiological responses of the patient to the odors are then measured via EEG. In the proposed study, a framework was established in which the suggested TQWCM images and spatial analysis techniques, CSP and CMTS, were fused at the feature level utilizing a transformer encoder. All features and the sub-band signals associated with TQWCM were fed to the encoder model as distinct tokens. The results were observed to be a strong approach in studies applied using only EEG and were determined as the highest success obtained with the dataset employed in the study. The imbalance in odor presentation (3:1 ratio of lemon to rose trials) is a limitation of the experimental design. Although trial order was randomized to reduce systematic bias, the more frequent lemon exposure could have led to greater habituation. This may partly explain why rose trials yielded higher classification accuracy, as participants might have remained more responsive to the less frequent rose scent. Our long–term objective is to translate the proposed framework into a practical tool. Future studies will therefore explore pruning the feature set and streamlining the transformer so as to preserve diagnostic performance while further reducing computational load. In addition, in future work, we plan to apply techniques such as explainable artifical intelligence (XAI) to further interpret which specific characteristics are driving the model's decisions.

## Data Availability

The original contributions presented in the study are included in the article/supplementary material, further inquiries can be directed to the corresponding author.
